# The heritability of *Nematodirus battus* fecal egg counts

**DOI:** 10.1017/S0031182022000014

**Published:** 2022-04

**Authors:** Saeid Nikbin, Fazel Almasi, Dalal Alenizi, Caitlin Jenvey, Sarah Sloan, Sarah Preston, David Piedrafita, Nicholas Jonsson, Michael Stear

**Affiliations:** 1 Department of Animal, Plant and Soil Science, Agribio, La Trobe University, Bundoora, Victoria 3086, Australia; 2 School of Science, Psychology and Sport, Federation University Australia, Ballarat, Victoria 3353, Australia; 3 Institute of Biodiversity, Animal Health and Comparative Medicine, University of Glasgow, Garscube Campus, Bearsden Road, Glasgow, Scotland G61 1QH, UK

**Keywords:** Bayes, heritability, Monte Carlo Markov Chain, nematodes, *Nematodirus battus*, sheep

## Abstract

Although *Nematodirus battus* is a serious threat to the health and survival of young lambs, there are few options to control this parasite. Bayesian Monte Carlo Markov Chain modelling with a zero-inflated Poisson distribution was used to estimate the heritability of egg counts in both June and July for each of five consecutive cohorts of 200 Scottish Blackface lambs. In one of the 10 analyses, the results failed the diagnostic tests. In seven of the analyses, there was no convincing evidence that the variation in egg counts was heritable. In the 2 years of high infection, the heritability was approximately 0.4 in June but the estimates lacked precision and the 95% highest posterior density credible intervals ranged from just above zero to 0.7. Selective breeding for resistance to *N. battus* will be difficult because genetically resistant or susceptible lambs cannot be consistently identified by phenotypic markers.

## Graphical abstract

**Figure:**
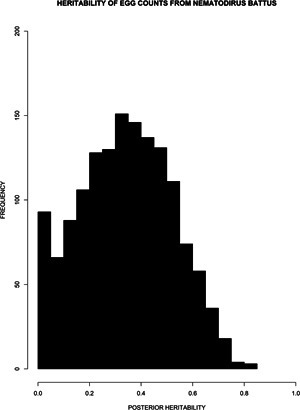

## Introduction

Gastrointestinal nematode infections are the most devastating disease of grazing sheep. Their estimated costs exceed $400 million every year in Australia (Lane *et al*., 2015), are about 100 million GBP per year in the UK (Nieuwhof and Bishop, 2005) and at least 400 million euros in the European Union (Morgan *et al*., 2013). Essentially, all grazing sheep are exposed to infection by multiple species of parasitic nematodes. The distribution of different species of nematodes is largely determined by their moisture and temperature requirements (Taylor *et al*., 2007). The major parasites in temperate climates are from the genera *Teladorsagia*, *Trichostrongylus* and *Nematodirus* while *Haemonchus* dominates in tropical and subtropical climates (Taylor *et al*., 2007).

Infected animals eat less, protein is digested less efficiently, protein is lost through immune-mediated breaches in the epithelium and protein is diverted away from meat and wool production to tissue repair and immune responses (Stear *et al*., 2003). Consequently, nematode infection causes a relative protein deficiency (Stear *et al*., 2003). Mixed infections are more pathogenic (Parkins *et al*., 1990); partly because protein lost in the abomasum cannot be resorbed efficiently in the small intestine when the small intestine is infected with nematodes. For *Haemonchus* species, the protein deficiency is exacerbated by anaemia.

There are several species of *Nematodirus* that infect sheep: *N. spathiger*, *N. filicollis* and *N. battus* are the most common *Nematodirus* species in the UK (Taylor *et al*., 2007); *N. spathiger*, *N. filicollis*, *N. abnormalis* and *N. helvetianus* have been reported in Australia (Beveridge and Ford, 1982). In addition, we have recently observed eggs that appear identical to the morphologically distinctive *N. battus* (unpublished observations) in samples from Merino sheep grazing in western Victoria, Australia.

*Nematodirus* eggs are larger than the eggs of other strongyle species, can be readily distinguished under the microscope and are usually reported separately from the other strongyles (Taylor *et al*., 2007). The morphologically distinctive eggs of *N. battus* are usually counted separately from strongyles and other *Nematodirus* species. *Nematodirus* larvae develop within the egg and do not hatch until the third larval stage (Taylor *et al*., 2007). Fecundity is lower for *Nematodirus* species than for other nematode taxa, with females producing only 20–30 eggs per day compared to thousands of eggs per day for female *H. contortus.* Despite the low fecundity, worm burdens in excess of 200 000 *Nematodirus* have been recorded (Brunsdon, 1967).

There is a widespread belief that *Nematodirus* species are less pathogenic than other species of nematodes but there is no published evidence for or against this assumption. Pathogenicity has been quantified for only *N. battus* and this species is extremely pathogenic and is capable of killing thousands of lambs when climatic conditions produce mass hatching of infective larvae (Taylor *et al*., 2007).

Nematode infections need to be controlled for sustainable and profitable farming. Currently, anthelmintics are the predominant method of nematode control but this method is threatened by increased drug resistance in nematode populations (Playford *et al*., 2014; Preston *et al*., 2019). Alternative methods of parasite control are urgently needed; they include the use of genetically resistant animals, the selective breeding of parasite-resistant and productive sheep, vaccination, nutritional supplementation, grazing management and the use of nematophagous fungi (Stear *et al*., 2007). Currently, there are no reliable vaccines against nematodes in cool temperate climates such as the UK and southern Australia (Britton *et al*., 2020). Nutritional supplementation is effective but usually too expensive and grazing management is often impractical. Biological control has promise but requires regular and infeasible handling of sheep. In contrast, selective breeding is cheap, effective and is being practiced by an increasing number of farmers.

At present, sheep are not being bred directly for resistance to *Nematodirus* species in general or for *N. battus* in particular. The heritability determines the rate of response of a trait to selection and determines the feasibility of selective breeding (Stear *et al*., 2001). There are no estimates for the heritability of traits in sheep related to resistance to *N. battus*. Previous estimates of the heritability of other *Nematodirus* species egg counts have come from the UK and New Zealand and were from 0.05 to 0.09 in British Blue Faced Leicester cross lambs at 10, 14 18, 22 and 26 weeks of age (Wolf *et al*., 2008). In British Texel lambs, the weighted average heritability was 0.38 (Bishop *et al*., 2004). In New Zealand Romney sheep, the heritability estimates were 0.15 and 0.26 (Morris *et al*., 2004). In addition, heritabilities for *Nematodirus* species of 0.17 and 0.21 in Coopworth sheep and 0.17 and 0.19 in Romney sheep of different ages have been reported (McEwan *et al*., 1995). These estimates were obtained from general linear models using transformed egg counts.

*Nematodirus battus* egg counts follow a zero-inflated negative binomial distribution (Denwood *et al*., 2008). One explanation is that lambs fall into two groups. One group contains animals which have not been exposed to nematode infection and all animals in this group have an egg count of zero. The other group consists of animals which have been infected and the number of eggs produced by this group follows a negative binomial distribution. Animals which have recently been exposed and are still within the prepatent period will be part of the first group. Egg counts from a population that contains both groups will follow a mixture of two distributions. Statistical models that assume appropriate distributions give more reliable estimates and software for the analysis of mixture distributions is now available in widely used statistical systems such as R, SAS and Stata. Egg counts in dairy cattle have been analysed as a mixture distribution (Nødtvet *et al*., 2002). Consequently, the aim of this paper was to use a mixture model to estimate the heritability of *N. battus* egg counts and assess the possibility of selectively breeding sheep for resistance to *N. battus* infection.

## Materials and methods

### Animals

The sheep were purebred Scottish Blackface from a commercial upland flock in the Strathclyde region near Lochwinnoch, Scotland, UK. Lambing took place over a 6-week interval from April to May. Each year, for 5 consecutive years from 1992 to 1996, a cohort of 200 lambs was sampled (Strain *et al*., 2002). The lambs were the offspring of 39 sires and 496 dams. To minimize inbreeding, rams were purchased privately or at sales, rather than being selected from within the flock. Lambing took place on three fields that had previously hosted lambs and were known to be contaminated with nematode larvae. The flock contained about 2000 ewes and each year, in order to monitor lambing, the farmer moved pregnant ewes to these fields, which were near the farmhouse, from outlying areas of the farm. The lamb populations were enriched with twins because twin-bearing ewes were preferentially moved and the number of ewes was adjusted to produce 200 lambs.

### Parasitology

Standard parasitological procedures were used to estimate the number of nematode eggs in the feces (Bishop *et al*., 1996). Briefly, feces were collected from the rectum every 4 weeks starting when the lambs were 4 weeks old. The numbers of *N. battus* eggs in a sample of 3 g were counted by a modified McMaster technique. Each egg counted represented 50 eggs per gram in 1992, 1993 and 1994. In 1995 and 1996, each egg counted represented 12.5 eggs per gram. All lambs were treated with a broad-spectrum anthelmintic (albendazole sulphoxide), given at the recommended dose rate of 5 g kg^−1^ body weight, based on the weight of the heaviest lamb at the time of treatment, after collection of each fecal sample. There was no resistance to albendazole within this flock, according to fecal egg count reduction tests. The samples collected from 4-week-old lambs included many lambs that did not provide a fecal sample, while the samples from lambs older than 12 weeks contained very few *N. battus* eggs. Consequently, this study concentrates on lambs that were on average 8 and 12 weeks old. In addition to the eggs from *N. battus*, there were eggs from other *Nematodirus* species and from additional strongyle species, predominantly *T. circumcincta* (Stear *et al*., 1995*a*, 1997*a*, 1997*b*, 1998, 1999; Stear and Bishop, 1999; Bishop and Stear, 2000).

### Analytical strategy

Initially the number of *N. battus* eggs counted from each lamb in the 10 cohorts of lambs were analysed in two generalized linear mixed models with the Glimmix procedure in SAS (SAS Institute Cary, North Carolina, USA). A separate model was used for each month because previous analyses have shown that the heritability of fecal egg count increases with time as lambs mature and the immune response develops (Bishop *et al*., 1996; Stear *et al*., 1997*a*). Previous analyses using both Bayesian and Maximum likelihood procedures had shown that *N. battus* egg counts in each cohort conformed to a zero-inflated negative binomial distribution (Denwood *et al*., 2008). The parameters of the best-fitting negative binomial distribution varied among the cohorts as did the extent of zero-inflation. The negative binomial distribution fitted to the combined dataset was able to capture all the observed variation in egg count. In other words, the variation among animals in the combined dataset was captured by the negative binomial distribution. The SAS code is in Supplementary file S1.

Fitting a single negative binomial distribution to a population that combines cohorts with different zero-inflated negative binomial distributions could lead to incorrect estimates of genetic parameters because animals that have not been exposed will falsely appear genetically resistant because they have egg counts of zero. There is no easy way within a combined analysis to set different parameters for each component of the mixture distribution in each cohort. In addition, a combined analysis is only appropriate if the trait is the same in all cohorts. It is possible that the immune response is effective only when the number of parasites exceeds a threshold. Therefore, egg counts in cohorts with a high intensity of infection could be measuring a different trait than egg counts in cohorts with a low intensity of infection. Consequently, we analysed each cohort separately. The R code for the June 1995 cohort is in Supplementary file S2. The other cohorts were analysed with identical models except for the number of iterations.

Animal models offer several advantages for the estimation of genetic parameters (Lynch and Walsh, 1998). The FMM procedure can analyse zero-inflated negative binomial distributions but cannot fit an animal model. The MCMCglmm package (Monte Carlo Markov Chain generalized linear mixed model) package in R (Hadfield, 2010) can fit an animal model but fits a zero-inflated Poisson rather than a zero-inflated negative binomial distribution. In the Poisson family of distributions, the variance is equal to the mean (*μ*), while in the negative binomial family of distributions, the variance is equal to *μ* plus (*μ*^2^/*k*), where *k* is an inverse index of overdispersion (Stear *et al*., 1998). As *k* increases, the negative binomial distribution becomes more similar to the Poisson.

### Statistical analysis

The univariate procedure in SAS was used to determine summary statistics (mean and variance) and draw histograms.

A generalized linear mixed model was fitted using the Glimmix procedure (Generalized Linear Mixed Model) in SAS. The analyses were done separately for each month but combined the data across all years. This model assumes that
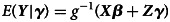


*E*(***Y*|*γ***) is the expectation of the egg counts conditional upon the random effects ***γ***, *g*() is a differentiable monotonic link function and *g*^−1^() is its inverse. ***X*** is an incidence matrix for the fixed effects ***β*** while *Z* is the design matrix for the random effects ***γ***. There were five fixed effects: year of birth, sex, birthtype, field and age which was fitted as a covariate. There were two random effects: sire and dam nested within sire. A negative binomial distribution was fitted with a log link function.

Proc FMM in SAS was used to determine the extent of zero-inflation in the observed values over the Poisson distribution while MCMCglmm was used to estimate the variance components for animal, dam and the residual, while sex, field and year were fitted as fixed effects. The heritability of *N. battus* egg counts was estimated as the proportion of the phenotypic variance accounted for by the variance in animal effects. The phenotypic variance was estimated as the sum of the animal, dam and residual variances. The dam variance includes both maternal genetic effects and maternal common environmental effects. These effects cannot be separated unless the dataset contains samples from the same mothers at different lambing times. The estimated heritability of *N. battus* egg counts is conditional upon our estimates of infection, conditional on the fixed effects of age (in days) as well as field and ignoring stochastic Poisson noise.

The MCMCglmm package handles overdispersion in an additive rather than multiplicative fashion. The residuals in a Poisson model are assumed to be independently and normally distributed with an expectation of zero. The residual variance models any overdispersion and a residual variance of zero implies that the response conforms to a standard Poisson. The default log link was used.

Initially, an Inverse Wishart prior was used for the variances and a normal prior for the fixed effects. The variance parameter (*n*) was set to 2. Each analysis was run for 1 500 000 iterations and the first 20 000 iterations were discarded (burnin). Every 500th iteration was stored. This gave similar results to keeping every 10th iteration but required less time to run. A diagonal variance structure was used because the covariance between the zero-inflated distribution and the Poisson distribution cannot be estimated. The inverse gamma prior is the default option in MCMCglmm but our initial analyses showed that the value of the prior determined the value of the heritability estimate. We therefore used parameter expansion with a proper Cauchy prior (Hadfield, 2010). With the location parameter set to zero and scale parameters at 1, 10 or 100, the heritability estimates differed by <0.02 and were essentially unchanged. Consequently, a location parameter of 0 and a scale parameter of 1 were used to analyse the data. The number of iterations was set to 150 000 or 1 500 000 with lags of 100 or 1000 respectively. The burnin was set to 2000. Thinning was set to 100 or 1000; i.e. only 1 in every 100 or 1 in every 1000 samples was retained. These parameter settings produced acceptable autocorrelations of <0.1 and effective sizes >1000. The MCMCglmm and coda packages also provide stationarity and half-width tests (Heidelberger and Welch, 1983; Koç *et al*., 2013). The parameter settings were chosen to ensure that these tests were passed.

MCMCglmm constrains the estimates of the variance components to be greater than zero. Consequently, testing the significance of the location parameters of the posterior distribution against zero is uninformative (Walter *et al*., 2018). The 95% highest posterior credible interval (HPD) was used to estimate the uncertainty in our estimate of the heritability. We estimated the variation among our estimates by repeating the analyses of the June 95 and June 96 samples 10 times and calculating the standard error of the mean. This measures the Monte Carlo noise. In addition, we ran each model with and without the animal effect and measured the deviance information criterion (DIC) for each model.

Two additional checks were used to ensure that the variance estimates were not unduly influenced by the choice of prior. A new pedigree file was created by allocating lambs sequentially to sires. The first lamb in numerical order was allocated to the first sire in numerical order, the second lamb was allocated to the second sire and so on. Each lamb was given a distinct dam. This has the same effect as randomization but the coding is simpler. In addition, 10 files were created using the RAND function in SAS to randomly assign sires to lambs. Each lamb was given a distinct dam. Each file was then analysed by MCMCglmm. Reshuffling the parents is expected to create a heritability close to zero if the estimate is not inflated by the prior.

## Results

**Fig. 1.**
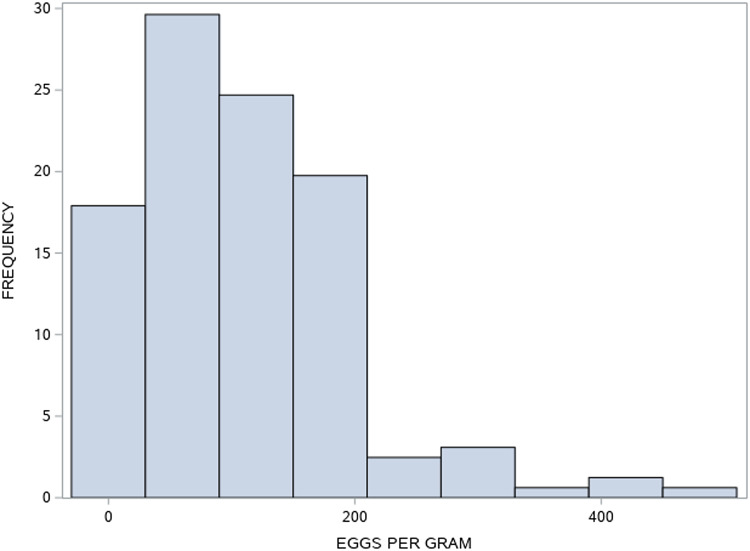
The distribution of *Nematodirus battus* egg counts in June 1992.

*Nematodirus battus* egg counts varied from 0 to 2000 eggs per gram with an arithmetic mean of 216 eggs per gram. The mean number of eggs per gram and the extent of zero-inflation over the negative binomial are shown in Table 1. The distribution for each cohort was different and the egg counts for June 1992 are illustrated in Fig. 1. Most lambs had relatively low egg counts but a small proportion had quite high counts. A disproportionate number of eggs therefore came from a small number of hosts.

**Table 1.** Number of lambs, time of sampling, zero-inflation relative to the Poisson distribution, fecal egg count (eggs per gram) for *Nematodirus battus*

Year	Month	Number of lambs	Zero inflation (%)	Mean egg count (epg)
1992	June	162	18	98
1992	July	148	13	15
1993	June	177	25	131.5
1993	July	189	66	25
1994	June	159	47	116.5
1994	July	161	46	127.5
1995	June	169	2	470
1995	July	186	30	40.5
1996	June	161	7	280
1996	July	177	46	45

Generalized linear mixed modelling with the GLIMMIX procedure in SAS on the June samples showed that the effects of year (*P* < 0.0001), field (*P* < 0.0001) and age (*P* < 0.0001) were significant but not the effects of sex (*P* = 0.655) or birth type (*P* = 0.367). The estimates of variance components were 0.009 ± 0.015 (mean ± standard error) for sire which was not significantly different from zero and 0.246 ± 0.058 for dam which was highly significant based on the size of the standard error relative to the estimate. The scale parameter was 0.381 ± 0.051. Analyses of zero-inflation with the Genmod and FMM procedures both indicated that there was no zero-inflation over the negative binomial for the combined dataset in June.

The initial generalized linear mixed analysis with the GLIMMIX procedure in SAS on the July samples did not converge. A subsequent analysis without dam showed that the effects of year (*P* < 0.0001) and field (*P* = 0.018) were significant although not the effects of age (*P* = 0.453), sex (*P* = 0.088) or birth type (*P* = 0.125). The estimates of variance components were 0 for sire and 1.671 ± 0.115 for the scale parameter. Analyses of zero-inflation with the Genmod and FMM procedures both indicated that there was no zero-inflation over the negative binomial for the combined dataset in July.

The egg counts for each cohort in each month were not well described by a negative binomial or a Poisson distribution because they had an excess of zeros above the numbers predicted by these distributions. For example, 29 of the 162 lambs in the June 1992 sample had egg counts of zero. They showed zero-inflation of 18% compared to the relevant Poisson distribution and zero-inflation of 2.5% compared to the best-fitting negative binomial distribution.

The Poisson and zero-inflated Poisson models fitting sire, dam, sex, field and age to the June 1992 cohort had Bayes Information Criteria (BIC) of 13 235 and 7427 respectively, indicating that the zero-inflated model was preferable. Therefore, a zero-inflated mixture model was used to analyse the data. As the extent of zero-inflation differed among months of sampling, each month was analysed separately.

**Fig. 2.**
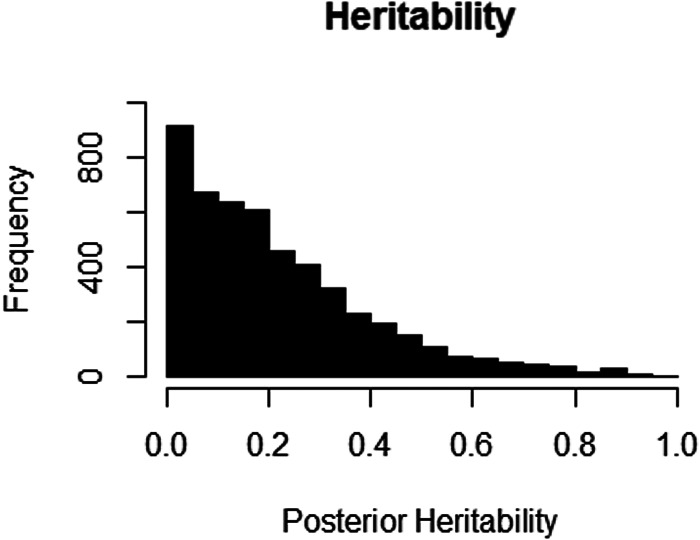
The distribution of posterior heritability estimates for the July 1994 samples. There was no indication in this cohort that egg counts were significantly different from zero.

**Fig. 3.**
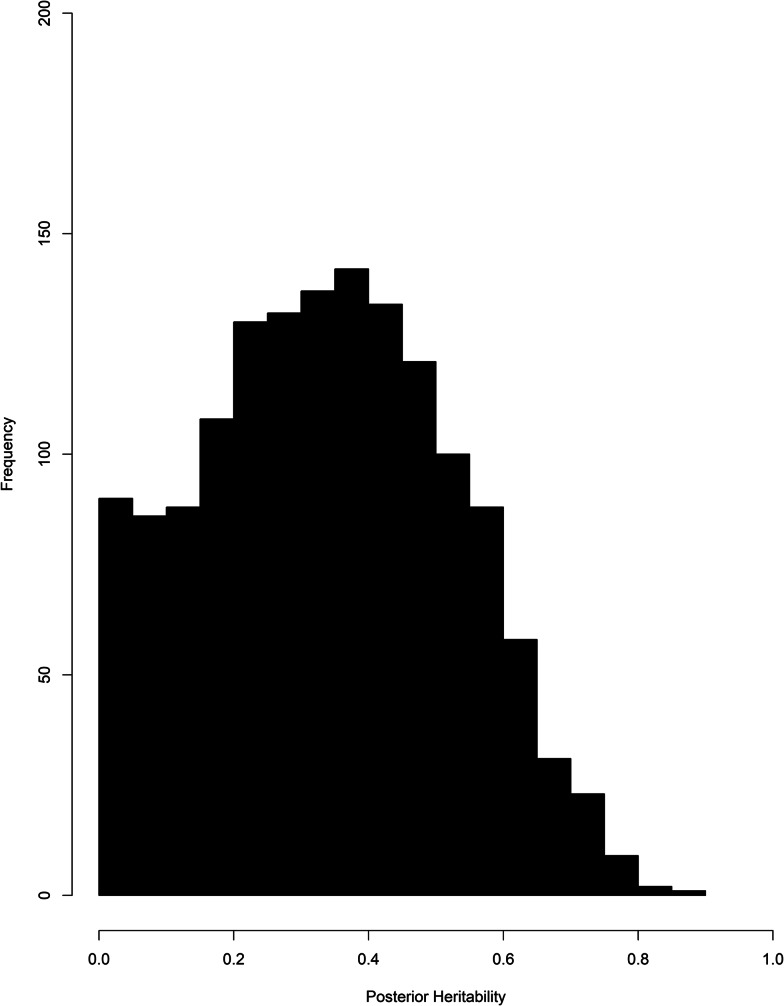
The distribution of posterior heritability estimates for the June 1995 samples. The unimodal and nearly symmetric distribution suggests that the true heritability is significantly different from zero.

The number of eggs counted in each of the 10 cohorts was analysed by a Bayesian Monte Carlo Markov Chain procedure in the R package MCMCglmm with a zero-inflated Poisson model and included the effects of animal, dam, age, sex and field. The results fell into three groups. The first group contained the results from one cohort (June 1994). The distribution of animal effects was left skewed but the model without animal effects failed the diagnostic tests because of high autocorrelations despite running the model for 5 000 000 iterations. The results from this model were therefore unreliable and were not considered further. The second group contained seven cohorts. Here the mean of the posterior distribution of the heritability differed from the median by at least 0.04, the distribution of animal effects was asymmetrical and dropped off from a peak near zero. DIC in the model without the animal effect was very similar to the DIC in the more complete model with the effect of animal. The pairs of values were (575.2, 575.4), (204.3, 203.9), (1354.4, 1354.2), (358.8, 357.5), (548.7, 551.7), (754.4, 758.0) and (659.0, 662.5) for June 1992, July 1992, June 1993, July 1993, July 1994, July 1995 and July 1996 respectively. In these seven cohorts, there was no convincing evidence for an effect of animal and hence no evidence that the variation in egg counts was heritable. Figure 2 illustrates the posterior distribution of animal effects for one cohort in this group. The third group contained two cohorts (June 1995 and June 1996). In these two cohorts, the mean and median of the posterior distribution were identical. The posterior distributions of animal effects were symmetrical with a peak well away from zero. In addition, the models without animal effects showed a substantially different DIC than the models with animal effects. The DIC were 1193.6 and 757.4 in June 1995 and 1058.2 and 663.7 in June 1996. Figure 3 illustrates the posterior distribution of animal effects for one of the two cohorts. These were the two cohorts with the highest mean egg counts.

The means of the posterior distribution of heritability, the HPD and an estimate of Monte Carlo variation are given in Table 2. The mean heritability (highest posterior credible interval) of *N. battus* egg counts was 0.43 (0.0000008–0.72) and 0.37 (0.0000006–0.72) in June 1995 and June 1996 respectively. The variation due to the Monte Carlo procedure (Monte Carlo noise) was estimated as the standard deviation of the mean in 10 replicates and was 0.003 in both June 1995 and June 1996.

**Table 2.** Time of sampling, estimated heritability of *Nematodirus battus* egg counts, 95% highest posterior density credible intervals (HPD) for one analysis and Monte Carlo variation (standard error of the mean of 10 replicated analyses)

Year	Month	Heritability (mean of posterior density)	Heritability (median of posterior density)	HPD	Monte Carlo variation (sem)
1992	June	0.15	0.10	0.00000020–0.48	–
1992	July	0.22	0.14	0.00000027–0.73	–
1993	June	0.26	0.21	0.00000072–0.67	–
1993	July	0.19	0.12	0.00000004–0.65	–
1994	June	DNC	DNC	–	–
1994	July	0.21	0.15	0.000000038–0.62	–
1995	June	0.43	0.43	0.00000078–0.72	0.003
1995	July	0.20	0.16	0.0000021–0.54	–
1996	June	0.37	0.37	0.00000055–0.72	0.003
1996	July	0.21	0.13	0.00000000046–0.69	–

DNC: There was an unacceptably high autocorrelation and the result was not used.

Figures S1–S3 show the trace and the density for the animal effects (Fig. S1), the dam effects (Fig. S2) and the residuals (Fig. S3) in June 1995. The traces are time series of each parameter as the MCMC iterated and the absence of a trend indicates that the analysis has converged. The density plot is a smoothed histogram of the posterior estimates of each parameter.

In order to test whether the heritability estimates could be an artefact of the analytical procedures, the pedigrees were shuffled and the data reanalysed. When the lambs were assigned to rams sequentially, the median heritability estimates for June 1995 and June 1996 were only 0.03 and 0.04. When sires were randomly allocated to lamb, the 10 populations had median heritabilities ranging from 0.032 to 0.250 with a mean of 0.084 and a standard error of the mean of 0.020. These results indicate that the choice of prior did not unduly influence the estimated heritability and that median heritabilities up to 0.250 could arise by chance alone.

## Discussion

Generalized linear mixed modelling with a negative binomial distribution was used to provide preliminary estimates of fixed and random effects. However, previous research (Denwood *et al*., 2008) has shown that the distribution of *N. battus* in each month is zero inflated. The extent of zero inflation and the parameters of the negative binomial distribution differed among all 10 cohorts from the 5 years. Consequently, each month was analysed separately. A Bayesian Monte Carlo Markov Chain procedure was used to estimate the heritability of fecal egg counts from this nematode species.

The posterior distribution of the animal effects was truncated at zero. The peak of the posterior distribution was close to zero in seven analyses and the distribution was right skewed. There was no evidence in these populations for heritable variation in egg counts. We therefore assumed that the positive heritability estimates in seven populations were generated by constraining the estimates of the animal effects to be above zero. In contrast, the two populations with high heritability estimates appeared symmetrical with means and medians well above zero. These two populations with the highest intensity of infection had heritability estimates of about 0.4. However, these estimates were not very precise and the 95% highest posterior density credible intervals were quite wide ranging from just above zero to about 0.7.

Previous analyses have indicated that resistance to *Teladorsagia circumcincta* develops quite slowly and heritabilities are not significantly greater than zero in lambs below 12 weeks of age (Bishop *et al*., 1996; Stear *et al*., 1997*a*). In contrast, our heritability estimates for *N. battus* were close to 0.4 in 8-week-old lambs in the 2 years of high infection. One explanation is that the immune mechanisms underlying resistance to *N. battus* and *T. circumcincta* are not the same. The immune mechanisms underlying resistance to *T. circumcincta* have been described (Stear *et al*., 1995*b*) but the immune mechanisms underlying resistance to *N. battus* are unknown although it is clear that lambs do mount an immune response (Israf *et al*., 1997). If relatively high infection rates are necessary to trigger effective immune responses, this would explain why the genetic variation was only apparent in years of high infection.

Detailed analyses of egg counts following nematode infections have shown that genetic variation accounts for much of the variation among animals within a flock (Bisset *et al*., 1992; McEwan *et al*., 1995; Bishop *et al*., 1996; Morris *et al*., 1996; Stear *et al*., 1997*b*; Woolaston and Piper, 2010). The low estimates of heritability for *N. battus* egg production in most populations examined in this study suggest that other sources of variation are important but these have still to be identified.

Strongyle egg counts follow a negative binomial distribution (Stear *et al*., 1998) and this distribution could arise from a combination of Poisson variation in the egg counting process and an underlying lognormal or gamma distribution (Hunter and Quenouille, 1952). If this explanation is applicable to *N. battus*, with the additional complication of zero-inflation, then fitting the animal model accounts for the variation among animals and the choice of a zero-inflated Poisson model provides an appropriate method to analyse *N. battus* egg counts in populations where the pedigree is recorded or estimated. Log normal variation among animal effects implies that genes act multiplicatively rather than additively, as commonly assumed in quantitative genetic theory.

Many natural parasite infections are likely to be zero-inflated (Basánez *et al*., 2004; Denwood *et al*., 2008; Ziadinov *et al*., 2010) but the use of zero-inflated distributions to analyse natural parasite infections is quite rare (Nødtvet *et al*., 2002; Sae-Lim *et al*., 2017). Now that free software is readily available, the use of these models should increase. Bayesian Monte Carlo Markov Chain procedures yielded significant heritability estimates even with relatively small sample sizes. However, the methods are not without problems. In particular, the number of variance structures that can be modelled is still quite limited.

The analysis of *N. battus* egg counts suggests that heritabilities will only be significant in years of high infection, which only occur when the weather promotes simultaneous hatching of many eggs (Taylor *et al*., 2007). This means that selective breeding for resistance to *N. battus* based on egg counts or the response to infection will be difficult because resistant animals can only be identified in some years. Unfortunately, there are no easy options to control *N. battus* if drug resistance continues to develop in parasite populations. Additional research is urgently needed.

In conclusion, *N. battus* egg counts are zero-inflated and a Bayesian Monte Carlo Markov Chain procedure was used to estimate the sources of variation and to determine genetic parameters. Young lambs showed moderate heritabilities for *N. battus* egg counts but only in years of high infection. The mechanisms underlying resistance to *N. battus* are unlikely to be identical to the mechanisms mediating resistance to *T. circumcincta*. Selective breeding for resistance to *N. battus* will be difficult because resistant animals can only be identified in years of high infection.

## Supplementary material

For supplementary material accompanying this paper visit https://doi.org/10.1017/S0031182022000014. click here to view supplementary material
