# Supplementary figures and images for: The heritability of Nematodirus battus fecal egg counts
Source: Parasitology. 2022 Jan 26;149(4):555–61. doi: 10.1017/S0031182022000014 (PMC10090607; doi:10.1017/S0031182022000014)

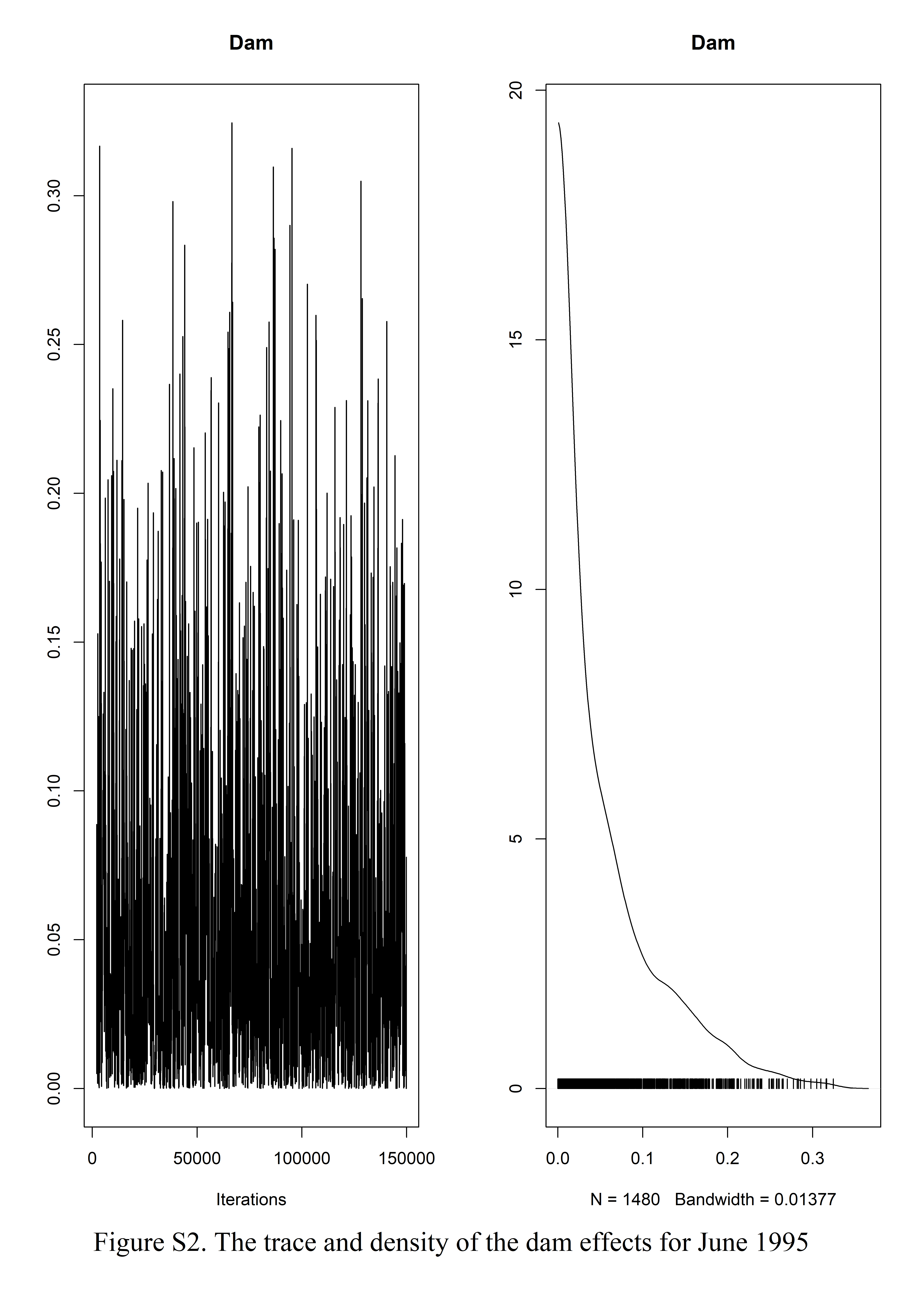

Supplement: Supplementary file 1 [file S0031182022000014sup.zip › S0031182022000014sup002.tif]

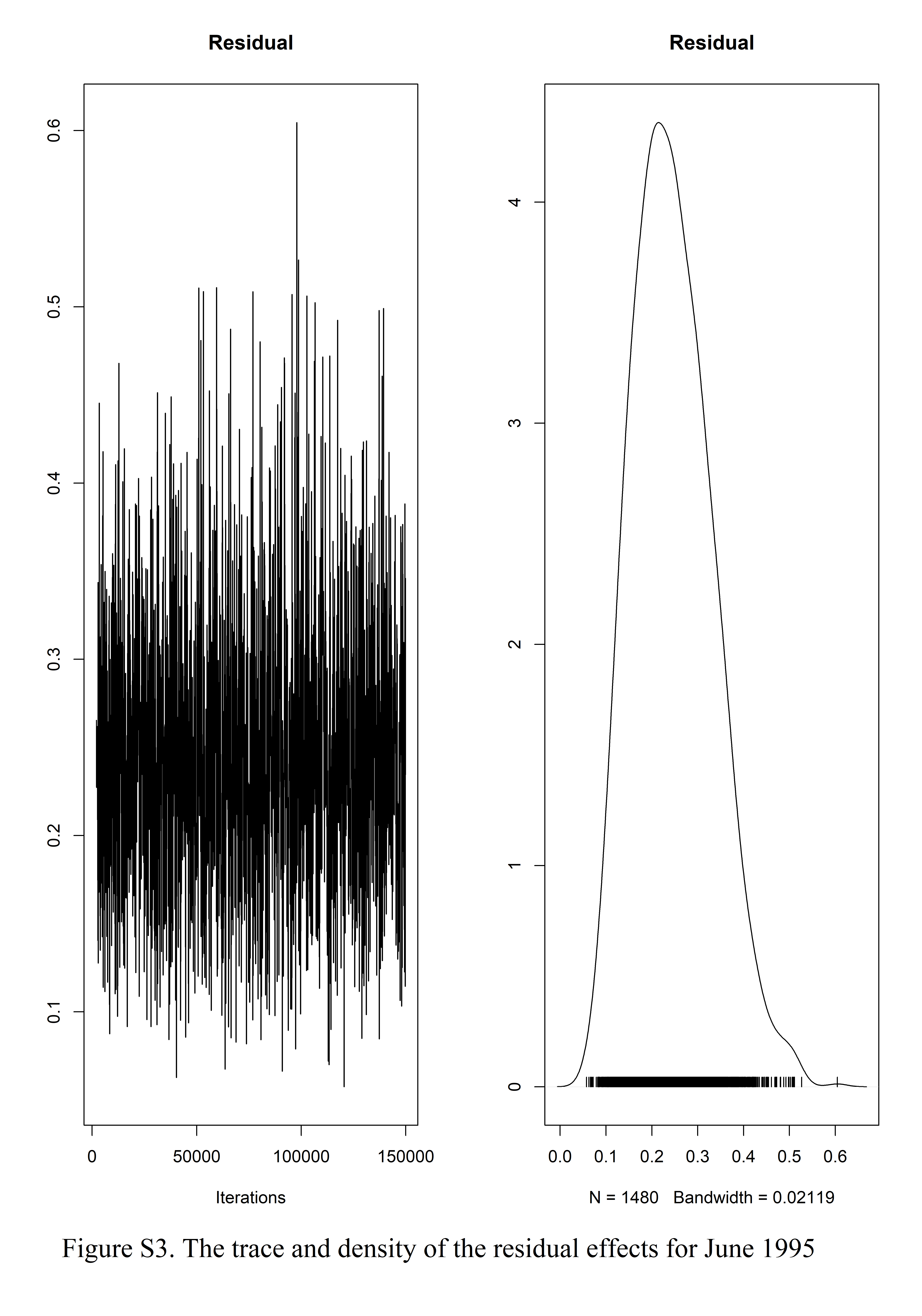

Supplement: Supplementary file 1 [file S0031182022000014sup.zip › S0031182022000014sup003.tif]
